# P-1827. Novel Evaluation System for *Aspergillus fumigatus* Interactions with Human Cells via Combination of Imaging and Electrical Impedance

**DOI:** 10.1093/ofid/ofae631.1990

**Published:** 2025-01-29

**Authors:** Shigeki Kakuno, Wataru Shibata, Takashi Umeyama, Yoshitsugu Miyazaki, Hiroshi Kakeya

**Affiliations:** Osaka Metropolitan University Graduate School of Medicine, Osaka, Osaka, Japan; Osaka City University, Osaka, Osaka, Japan; National Institute of Infection Disease, Tokyo, Tokyo, Japan; National Institute of Infectious Diseases, Shinjuku-ku, Tokyo, Japan; Osaka Metropolitan University, Osaka, Osaka, Japan

## Abstract

**Background:**

*Aspergillus fumigatus* is a major fungal pathogen causing pulmonary aspergillosis, an important disease associated with transplantation and immunosuppression. However, the interactions between *A. fumigatus* and human cells are not well understood. This study evaluated the interaction of *A. fumigatus* with THP-1 macrophages and A549 lung epithelial cells using fluorescence imaging and electrical impedance measurements to monitor fungal growth and cellular damage continuously.

**Figure 1.**
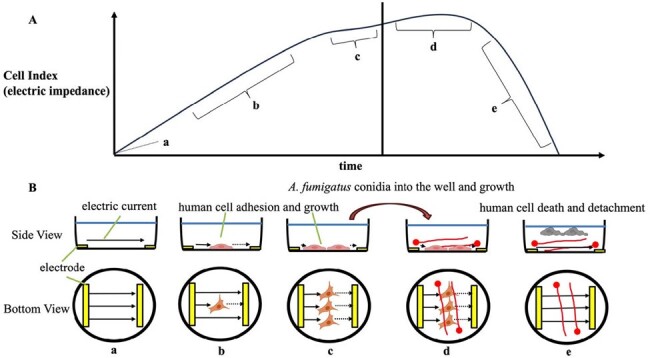
Schematic diagram of the relationship between electrical impedance and cell damage

**Methods:**

The experimental setup used xCELLigence eSight (Agilent) and a specialized 96-well plate with electrodes at the bottom to measure impedance changes as cells adhered or detached (Figure 1). THP-1 and A549 cells were cultured, then infected with fluorescent *A. fumigatus* conidia. In addition, antifungal drugs (micafungin, caspofungin, voriconazole, isavuconazole, amphotericin B) were added to this experimental system at broth microdilution method and tested.

**Figure 2.**
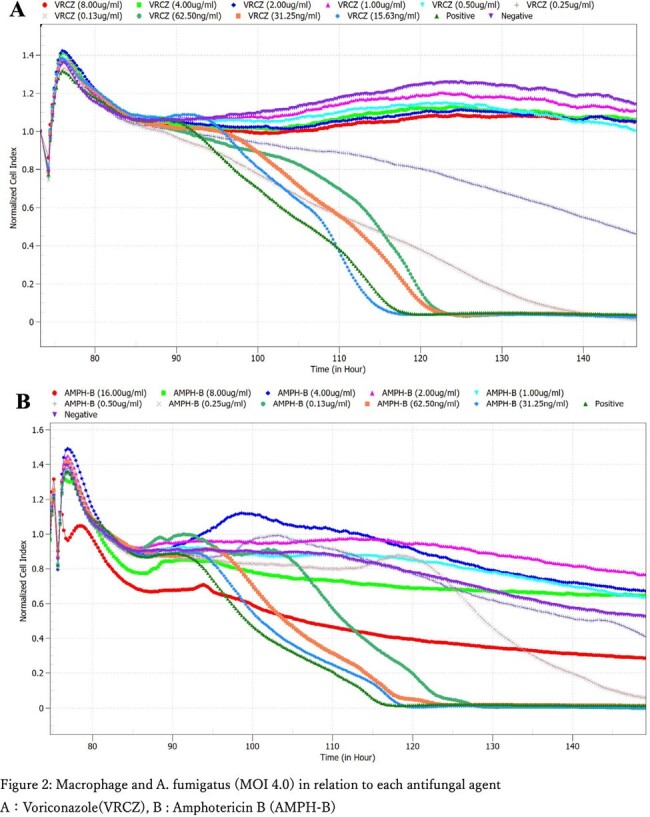
Macrophage and A. fumigatus (MOI 4.0) in relation to each antifungal agent A:Voriconazole(VRCZ), B : Amphotericin B (AMPH-B)

**Results:**

Results showed *A. fumigatus* filament growth leading to decreased impedance indicative of cell damage in both cell types, with faster damage at higher conidial loads. Fluorescence showed echinocandins inhibited hyphal growth but did not prevent impedance drops. Voriconazole and isavuconazole prevented impedance drops at clinically relevant concentrations (Figure 2A). Amphotericin B caused rapid impedance drops at high doses, suggesting direct cytotoxicity (Figure 2B).

**Conclusion:**

A549 epithelial cells survived longer than macrophages, suggesting they play a defensive role against *A. fumigatus*. Impedance changes correlated with visual antifungal effects for azoles and amphotericin B but not echinocandins. This system enables evaluating fungal-host interactions using different cell types and fungi. Combining impedance with fluorescence imaging provides a comprehensive and objective assessment of antifungal activity and cellular responses.

**Disclosures:**

**Hiroshi Kakeya, MD, PhD**, Asahikasei Phrma: Honoraria|GSK: Honoraria|MSD: Honoraria|Pfizer: Honoraria|Shionogi: Honoraria|Sumitomo Parma: Honoraria

